# Exploring factors associated with inflammation in stressed workers: a cross-sectional study

**DOI:** 10.1186/s12889-025-24754-1

**Published:** 2025-11-14

**Authors:** Akihiro Koreki, Hisateru Tachimori, Anna Kubota, Yoshiaki Kanamori, Manae Uchibori, Shiyori Usune, Akira Ninomiya, Akihiro Fujimoto, Kanako Inabe, Ryutaro Shirahama, Yasue Mitsukura, Hiroaki Miyata, Masaru Mimura, Mitsuhiro Sado

**Affiliations:** 1 Department of Neuropsychiatry, Keio University School of Medicine, Shinanomachi 35, Shinjuku-ku, Tokyo, 160-8582 Japan; 2 Center for Stress Research, Keio University, Tokyo, Japan; 3 Department of Psychiatry, NHO Shimofusa Psychiatric Medical Center, Chiba, Japan; 4 Department of Health Policy and Management, Keio University School of Medicine, Tokyo, Japan; 5 Endowed Course for Health System Innovation, Keio University School of Medicine, Tokyo, Japan; 6 Medical Headquarters, Eisai Co., Ltd, Tokyo, Japan; 7 Faculty of Science and Technology, Keio University, Kanagawa, Japan; 8 Center for Preventive Medicine, Keio University, Tokyo, Japan; 9 Health Center, Keio University, Tokyo, Japan

**Keywords:** IL-6, Inflammation, Occupational stress, Fatigue

## Abstract

**Background:**

Chronic occupational stress leads to physical and mental illnesses, highlighting the importance of effective stress management in modern societies. Recently, chronic stress-induced systemic low-grade inflammation has garnered increasing attention for its stress response and crucial pathological role in the development of both physical and mental illnesses. In this context, elevated salivary interleukin-6 (sIL-6) levels have been developed as measurable and non-invasive stress indicators. However, the factors associated with sIL-6 in stressed office workers, including the symptomatic manifestations of stress responses, have not been extensively investigated. Since direct measurement of inflammation is costly in routine stress management, identifying symptoms that reliably reflect inflammation could facilitate the development of more effective, inflammation-based stress management strategies.

**Methods:**

In this cross-sectional study, stressed office workers were recruited through a screening process using a Brief Job Stress Questionnaire. Saliva samples were collected to measure sIL-6 levels, and participants completed questionnaires addressing their occupational environment, symptomatic manifestations, and lifestyle. After excluding one participant owing to a medical history that could potentially affect sIL-6 levels, 128 stressed office workers were included in the analysis.

**Results:**

Our model-based analysis for symptomatic manifestation demonstrated that elevated sIL-6 was significantly associated with greater feelings of fatigue as well as relatively higher vigor. A separate model-based analysis for stressors revealed a significant association with higher qualitative job overload. Data-driven analyses further supported the finding that elevated sIL-6 was significantly associated with fatigue.

**Conclusions:**

Our findings suggest that the feeling of fatigue may reflect chronic stress-induced systemic low-grade inflammation in the body, highlighting the importance of self-monitoring fatigue for early intervention. Inflammation-based stress management holds promise in preventing both mental and physical illnesses in stressed office workers.

## Background

Occupational stress and consequent mental and physical illnesses are crucial issues worldwide. A survey of occupational stress-related mental health problems conducted by the Japanese government demonstrated that 8.8% of all workers required medical leave, and 4.1% had to permanently leave their companies [1]. The combination of occupational stress and individual vulnerability can induce various mental health problems, such as insomnia, adjustment disorders, depression [2–5], and even suicide [6]. In addition, these consequences can impair cognitive functioning, further harming individuals’ well-being and work performance [7]. Moreover, it can contribute to physical illnesses and, in extreme cases, sudden death [8, 9]. Therefore, managing occupational stress is essential for preventing detrimental consequences. However, current standard assessments primarily rely on questionnaires, which carry the risk of underestimating stress and lead to biased assessments because of their self-reporting nature. In contrast, growing evidence highlights stress-induced inflammation as a key contributor to both mental and physical health problems [10–15]. This suggests that workers may exhibit varying levels of inflammation regardless of their self-reported stress levels, underscoring the need for inflammation monitoring as a crucial component of effective stress management.

Stress is a broad concept classified into stressors and stress responses [16]. Stressors from the external environment pose dangers to humans and lead to stress responses to manage such threats. Stressors can be categorized as acute or chronic. Acute stressors include trauma and infection, whereas chronic stressors include repeated and prolonged exposure, work-related stress, isolation/loneliness, and caregiving [17]. According to the model presented by the National Institute for Occupational Safety and Health, occupational stressors such as excessive workload and interpersonal conflict lead to stress responses [2]. The hypothalamic-pituitary-adrenal (HPA) axis and sympathetic nervous system are activated during stress responses, which subsequently activate and dysregulate the immune system via various mechanisms, including reduced sensitivity to glucocorticoids, especially under chronic stress [10, 12, 14, 18–21]. These comprehensive physiological changes are adaptive reactions induced to manage acute dangers, but continuous and inflexible physiological changes lead to “wear and tear” damage to the body, including the brain [13].

Particularly, chronic stress-induced systemic low-grade inflammation as a bodily stress response has garnered increasing attention for its pathological role in the development of both mental and physical illnesses, such as insomnia/depression [22–24] and cardiovascular diseases [17]. A relationship between serum inflammation levels and occupational stress has been reported [20]. Therefore, managing low-grade inflammation through early intervention may have substantial preventive effects on various occupational stress-related illnesses. Interleukin-6 (IL-6) is a well-studied cytokine with a central role as a proinflammatory mediator [24]. Recently, salivary interleukin-6 (sIL-6) was proposed as a noninvasive and reliable biological stress marker [25, 26]. Saliva contains components derived from blood through passive diffusion or active transport, and correlations between IL-6 levels in plasma and saliva have been documented [25, 27]. It is induced by a chronically activated HPA axis and sympathetic nervous system, along with a decreased parasympathetic nervous system [19]. Furthermore, it is more stable than other salivary biological markers, such as amylase and cortisol, which are more vulnerable to sampling timing, and cardiological markers for stress responses, such as heart rate and heart rate variability, which are dramatically changeable and unsuitable for the assessment of chronic occupational stress [28–30].

Various occupational stressors can induce inflammation as a bodily stress response, ultimately resulting in conditions characterized by a range of symptoms, such as fatigue, insomnia, burnout, and depression. Notably, fatigue is suggested to be particularly associated with inflammation. Several studies investigating specific symptoms of depression have reported that inflammation indexed by C-reactive protein (CRP) was significantly associated with fatigue, sleep disturbances, and eating problems [31, 32]. Cohort studies have demonstrated that high CRP and IL-6 levels predict fatigue in childhood and adulthood [33, 34]. Intervention studies using IL-6 or IL-6 inhibitors have suggested that IL-6 plays a key role in inducing the subjective feeling of fatigue. Beyond mental disorders, significant associations between fatigue and inflammation have been consistently reported in various physical diseases [35–43].

Altogether, although inflammation due to bodily stress reactions in stressed workers is expected, the specific factors, including symptomatic manifestations of stress responses, associated with sIL-6 have not been extensively investigated. Notably, since direct measurement of inflammation is costly in routine stress management, identifying symptoms that reliably reflect inflammation could facilitate the development of more effective, inflammation-based stress management strategies. The management of low-grade inflammation can have substantial preventive effects against various diseases. Therefore, in the current study, our primary aim is to identify factors associated with sIL-6 in stressed office workers, including symptomatic manifestations. To focus on occupation-related psychological stress and minimize heterogeneity in work environments and potential stressors, we specifically targeted office workers.

## Methods

### General procedures

This cross-sectional observational study was conducted at the Keio University School of Medicine under the ethical guidelines set forth by the Declaration of Helsinki, approved by the ethics committee at Keio University School of Medicine (2021-1031-4), and registered at UMIN (UMIN000045528). All participants visited the University and provided written informed consent before participation. They were then asked to complete the following questionnaires in a quiet room without rushing. Subsequently, saliva was collected to assess sIL-6 and salivary immunoglobulin A (sIgA) levels. As with all procedures in this study, after these assessments, a subset of participants (not all) was asked to wear an Apple Watch Series 6 (Apple Inc., Cupertino, CA, USA) during sleep to monitor sleep quality over the week using a simple daily recording. An analysis of sleep structure has already been published [23] that demonstrated a significant association between sIL-6 levels and sleep quality. Therefore, the current analysis does not include data from the Apple Watch.

### Participants

Participants were enrolled via a web-based patient recruitment service (3 H medi solution: https://global-3 h.com/). This study focused primarily on occupation-related psychological stress, specifically targeting office workers to reduce heterogeneity in the work environment. Given the diversity of conditions, even among stressed office workers, we purposefully selected individuals experiencing high levels of stress, aiming to enhance the sensitivity to detect the direct relationship between the level of sIL-6 and their characteristics, thereby avoiding the general gradational relationship that stems solely from the overall severity. The inclusion criteria were as follows: (1) aged 20–65 years and full-time workers; (2) individuals who provided written informed consent to participate in this investigation; and (3) workers who experienced high levels of occupational stress. High-stress participants were identified based on a job demand-control model of occupational stress [44]. The initial screening specifically aimed to recruit stressed office workers and was conducted using two elements of the Brief Job Stress Questionnaire (BJSQ) [45]: job demand and job control. Individuals with scores exceeding the average scores of the Japanese population in both areas (2.14 for *quantitative job overload* and 2.53 for job control [46] were classified as stressed office workers. The exclusion criteria were as follows: (1) workers on leave; (2) night shift workers (10 pm–5 am); (3) workers involved in direct fieldwork/manufacturing processes in sectors such as manufacturing, construction, restaurants, mining, agriculture, forestry, and fishing; (4) individuals with a history of psychiatric conditions and/or current psychiatric illnesses; (5) individuals currently taking psychotropic medications; and (6) principal investigators and research team members deemed ineligible to participate in the study.

Notably, as this study was conducted during the COVID-19 era, enrolled participants were strictly assessed for potential infection based on body temperature and the presence of symptoms, revealing no risk of infection in this population.

### Saliva samples

Saliva samples were collected once, at the time of the participants’ visit to our university, to assess sIL-6 and sIgA levels. Saliva was deposited into test tubes using a straw, and the samples were immediately stored in a freezer. The samples were frozen and transported to LSI Medience Inc. (Tokyo, Japan) to analyze their saliva components. sIL-6 was measured using a chemiluminescent enzyme immunoassay, and sIgA was measured using an enzyme immunoassay. Ideally, the timing of saliva collection and participants’ conditions, such as their fasting status and recent physical activity, should have been standardized across participants to account for diurnal variation. However, due to scheduling constraints related to both participant availability and laboratory access, samples were collected between 10 a.m. and 5 p.m. The diurnal pattern of salivary IL-6 during waking hours has been reported to follow a characteristic trend: it peaks at awakening, drops significantly by 10 a.m., continues to decline steadily until reaching its lowest point around 1 p.m., and then gradually increases, with a marked rise from evening into the night [28].

In the current study, sIL-6 was set as the primary outcome for the objective stress marker because it directly reflects inflammation and higher levels of sIL-6 can be interpreted as stronger stress responses [26]. sIgA was also measured as a reference to reveal its relationship with sIL-6 in occupational stress, given that it has been proposed as another potential objective stress marker, and pro-inflammatory mediators, such as IL-6, stimulate B-cell activity for inducing immunoglobulin production. However, sIgA levels can fluctuate depending on whether the stress is acute or chronic, reportedly increasing under acute stress and decreasing under chronic stress [47–49].

### Questionnaires

A set of questionnaire-based assessments was designed to explore stress-related characteristics, all of which could be associated with bodily stress responses, including inflammation. To evaluate these aspects, the following questionnaires were administered (Table 1), alongside questionnaires for general characteristics and their habits: BJSQ [45] and Job Contents Questionnaire (JCQ) [50] for comprehensive occupational stress assessment, Dutch Work Addiction Scale (DUWAS) [51] for work addiction, WHO-Health and Work Performance Questionnaire Short Form (WHO-HPQ-SF) [52] for work performance, Pittsburgh Sleep Quality Index (PSQI) [53] and Epworth Sleepiness Scale (ESS) [54] for the assessments of sleep, Quick Inventory of Depressive Symptomatology (QIDS) [55] for depression, Multidimensional Assessment of Interoceptive Awareness (MAIA) [56] for the multifaced assessment of interoception, Mindful Attention Awareness Scale (MAAS) [57] for mindful level, and Satisfaction with Life Scale (SWLS) [58] for well-being.

**Table 1:** Unstructured questionnaires collected from participants

General information	age	(years old)
	sex	male, female, others
	educational years	(years)
	height and weight (BMI)	(kg/m2)
	smoking status	smoker, non-smoker
	marriage status	married, common-law marriage, unmarried, divorced, bereaved
	home environment	living alone, with a spouse, with spouse and children, with a partner, multi-generational
Lifestyle	**Dietary and drinking habits**	
	frequency of diet	one to two meals/day, three meals/day
	frequency of eating snacks	not at all-rarely, once a day, several times a day
	frequency of eating vegetables	not at all-rarely, sometimes, once a day, every meal
	frequency of drinking alcohol	no drinking, less than once a month, 2–4 times a month, 2–3 times a week, 4 or more times a week
	coffee consumption	not at all, less than 1 cup/day, 1 cup/day, 2–3 cups/day, more than 3 cups/day
	energy drink consumption	not at all, less than 1 cup/day, 1 cup/day, 2–3 cups/day, more than 3 cups/day
	green tea consumption	not at all, less than 1 cup/day, 1 cup/day, 2–3 cups/day, more than 3 cups/day
	red tea consumption	not at all, less than 1 cup/day, 1 cup/day, 2–3 cups/day, more than 3 cups/day
	**Daily activities**	
	frequency of exercise	not at all, once a month, once a week, several times a week, almost every day
	time spent using any devices for personal use	not at all/very little, 1 h a day or less, 1–2 h a day, 3–4 h a day, more than 1 h a day
	time spent playing games	not at all/very little, 1 h a day or less, 1–2 h a day, 3–4 h a day, more than 1 h a day
	types of light bulbs in bedrooms	incandescent light bulb, LED
	bath time	(minutes)
Work	**Position and experience**	
	job category	technical work, clerical work, system engineering, sales, others
	job position	manager level, not manager level
	years of employment	(years)
	years of current employment	(years)
	previous leave or absence	yes, no
	**Working style**	
	days of working	(days/week)
	working h/day	(h/day)
	time spent using computers	(h/day)
	rest time	(h/day)
	**Commuting style**	
	commuting time	(minutes)
	days of commuting	(days/week)
	telework	≥1 time/week or not

The BJSQ is one of the most widely used assessments in Japan and consists of four major parts [45]. Scoring in the present study was conducted using the most recent scoring system to ensure alignment in the direction of meaning across all measures [59]. A total of 57 items, each rated on a 4-point Likert scale, assess the following aspects. The first part evaluates eight domains related to subjective work-related stressors and environmental modifiers: quantitative job overload, qualitative job overload, physical demands, interpersonal conflict, job control, skill utilization, suitable job fit, and meaningfulness of work. The second part measures both physiological and psychological stress reactions. In particular, psychological stress is assessed across five facets: vigor, irritability, fatigue, anxiety, and depression. The third part evaluates perceived support from supervisors, coworkers, and family and friends. The fourth part assesses satisfaction with work and life. Each item is scored on a scale from 1 to 4 and averaged within each domain or facet, with lower scores indicating more negative evaluations (e.g., higher job overload, less job control or less meaningful work).

JCQ is one of the internationally standardized assessments for occupational environments [50]. A total of 22 items, each rated on a 4-point Likert scale, assess the following aspects. It is based on the demand–control–support model and consists of three core components: decision latitude (comprising skill discretion [score range: 12–48] and decision authority [2, 10, 12–46]), psychological demand (12–48), and social support (including supervisor support [4–16] and coworker support [4–16]). Lower scores generally reflect more negative evaluations, except for psychological demand, where a higher score reflects greater psychological strain. In addition, the Demand/Control ratio was calculated as psychological demand divided by decision latitude multiplied by 9/5, where higher scores reflect more adverse evaluations.

The DUWAS assesses an addictive attitude toward work, which is not directly captured by the BJSQ or JCQ but could influence occupational stress [51]. It primarily consists of 10 items, each rated on a 4-point Likert scale, evaluating two facets: working compulsively and working excessively, as well as their combined total score. Each item is scored on a scale from 1 to 4 and averaged within each facet, with higher scores indicating more negative evaluations. This questionnaire also includes an item assessing overwork, calculated as the difference between actual working hours and contracted hours. Although work-shift status was also evaluated, night shift workers were excluded from the present study. To avoid confusion, the work-shift item was not included in our analysis.

The WHO-HPQ Short Form (WHO-HPQ-SF) can estimate absolute and relative presenteeism, which reflects dysfunctions in work performance that may be stress-induced in this context [52]. The scale includes two items: one assessing the individual’s own work performance and the other assessing the average performance of their coworkers. Both items are rated on a scale from 0 to 10. Absolute presenteeism is calculated by multiplying the self-rated performance score by 10 (yielding a range of 0 to 100). Relative presenteeism is calculated by dividing the self-rated performance score by the coworker’s performance score (range: 0.25 to 2.0). Higher scores on both indices indicate better work performance.

The PSQI includes 18 items that assess multiple aspects of sleep, yielding a global score and seven subscales: subjective sleep quality, sleep latency, sleep duration, habitual sleep efficiency, sleep disturbances, use of sleep medication, and daytime dysfunction [53]. Each subscale score ranges from 0 to 3, and the global score ranges from 0 to 21. Higher scores indicate poorer sleep quality. Sleep quality and occupational stress are considered to be mutually influenced [23, 60].

The ESS is designed to assesses daytime sleepiness [54]. It consists of 8 items, each rated on a 4-point Likert scale, yielding a total score ranging from 0 to 24. Higher scores reflect greater levels of daytime sleepiness.

The QIDS assesses depressive symptoms across a broader range of aspects (16 items) compared to the depression subscale in the BJSQ [55]. The total score ranges from 0 to 27, with higher scores indicating greater severity of depression.

The MAIA subjectively assesses various dimensions of interoceptive functioning [56], which play a key role in monitoring and regulating bodily responses to stress [61], potentially modulating the association between stressors and stress responses. It consists of 32 items that contribute to eight subscales: noticing, not-distracting, not-worrying, attention regulation, emotional awareness, self-regulation, body listening, and trusting. Each subscale is scored from 0 to 5, with higher scores generally indicating greater interoceptive sensibility to internal bodily sensations [62].

The MAAS is a self-reported questionnaire that measures dispositional mindfulness, which is also recognized as a key factor in stress reduction [57]. It consists of 15 items, each rated on a 6-point Likert scale ranging from 1 to 6, resulting in a total score range of 15 to 90. Higher scores indicate greater levels of mindfulness.

The SWLS measures life satisfaction through five questions [58]. The total score is in the range of 5 to 35, and a higher score indicates better subjective well-being. This construct is evaluated within the framework of positive psychology, which emphasizes strengths and well-being rather than pathology. Life satisfaction could serve both as a protective factor that mitigates work-related stress, and as an outcome shaped by overall work experience.

Additional participant characteristics were assessed and grouped for our analysis based on the meaning of each item and the number of participants in each category. These unstructured questions, designed to capture more detailed information about participants’ work and lifestyle (including changes during the COVID-19 era), were developed to supplement items not covered by the structured questionnaires, as these factors can all be considered relevant to stress and sleep (for another published project [23]). In this study, we selected and analyzed variables that were presumed to have a more direct relationship with participants’ stress. These items were organized into three meaningful domains with subdomains: general information, lifestyle (dietary and drinking habits/daily activities), and work (position and experience/working style/commuting style). Specific beverage types were arbitrarily selected to reflect common preferences in Japanese culture in workplace contexts (Table 1).

### Statistics

#### Univariate analyses

To explore potentially significant factors, we first examined the association between sIL-6 levels and patient characteristics. Given the right skewness of sIL-6 and sIgA distributions, a log transformation was performed prior to all analyses. The normality of each variable was visually assessed using Q–Q plots. Welch’s t-test, or analysis of variance, was used for categorical variables. Pearson or Spearman correlations were calculated for continuous variables. In addition, Cronbach’s alpha was used to assess the internal consistency reliability of the questionnaires.

#### Variable selection and modeling

To avoid overly complex models with an excessive number of variables, we conducted the following analyses: two model-based approaches and one data-driven analysis. In all models, multivariate analysis was conducted with sIL-6 as the dependent variable and the potential factors as independent variables. Several items were recategorized and then included as categorical variables in the models, as their responses had fewer than five levels. Specifically, smoking status was categorized as either currently smoking or not; sleep duration was as short (< 7 h, corresponding to a score of 1 or higher) or not; and green tea consumption was grouped into three categories: none, 0–1 cup/day, and 2 or more cups/day, based on the distribution of participants across categories. The home environment variable was recategorized into multi-generational or not to reduce the number of variables in the model. Significant MAIA subscales were consolidated into a single factor based on principal component analysis. Since job overload measured by the BJSQ and psychological demand measured by the JCQ are conceptually similar, our model included only job overload as measured by the BJSQ. Multicollinearity was assessed using variance inflation factors (VIFs), with a maximum VIF value reported for each model. A p-value of less than 0.05 was considered statistically significant. Statistical analyses were performed using R software (4.3.2 or later) (R Core Team, Vienna, Austria).

1. Symptom-based analysis.
  This analysis aimed to identify symptoms that reliably reflect inflammation, to support inflammation-based self-monitoring, based on the assumption that symptomatic manifestations represent underlying inflammatory states resulting from stress responses. The model included physical stress and five psychological stress-related factors—vigor, irritability, fatigue, anxiety, and depression (measured by the BJSQ). Age, sex, BMI, smoking status, and the time of saliva sample collection were also included as covariate adjustments.
2. Stressor-based analysis.
  This analysis aimed to explore which factors are linked to inflammation, based on the assumption that inflammation may be caused by stressors and modulated by buffering factors. The model included work-related stressors (as measured by the BJSQ) to maintain consistency with the symptom-based analysis. Age, sex, BMI, smoking status, and the time of saliva sample collection were also included as covariate adjustments.
3. Data-driven analysis.
  This analysis aimed to complement the preceding analyses and to explore additional factors potentially associated with sIL-6 levels. Variables with a *p*-value < 0.1 in the univariate analyses were included in a multivariate analysis (a broader model). This relatively liberal threshold had been pre-specified in the study protocol for exploratory purposes, to minimize the risk of excluding potential confounders from subsequent modeling. Additionally, as a sensitivity analysis and to reduce the number of variables, another multivariate analysis was performed using only variables with a *p*-value < 0.05 in the univariate analyses (a narrower model).

## Results

### Characteristics

**Fig. 1:**
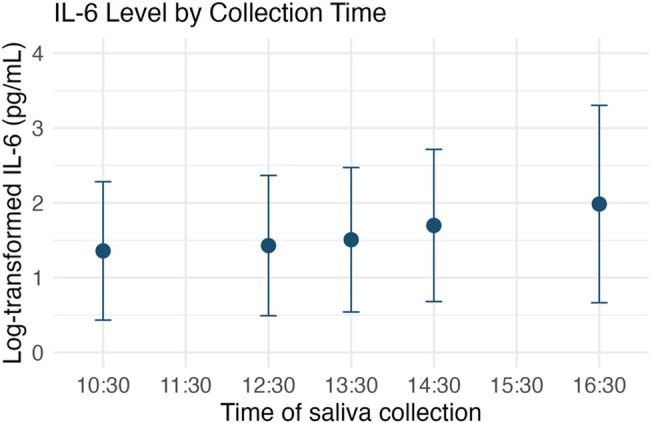
Higher sIL-6 levels were observed at later collection times (rho = 0.174, p = 0.0498)

In total, 130 participants were recruited. One participant withdrew consent, and one participant was excluded from the study because of receiving steroids, which affected the level of IL-6. Finally, the data of 128 participants (mean ± standard deviation [SD] age: 41.6 ± 10.9 years old; male/female: 60/68) were analyzed. Most participants (94.5%) worked 5 days per week for an average of 8.8 ± 1.3 h per day. A few participants (35.2%) reported telework on one or more days per week. As our inclusion criteria, they reported high occupational stress assessed by the BJSQ, including greater quantitative job overload (mean ± SD: 1.67 ± 0.50) and smaller job control (2.22 ± 0.56), which were more severe than the national average. While 109 participants were non-smokers and 2 had quit smoking, 17 were current smokers. Regarding saliva samples, their level of sIL-6 was median 4.02 [interquartile range: 7.10] pg/mL and sIgA 160 [96] µg/ml. No difference between sampling times (morning and afternoon) (*p* = 0.148 in sIL-6 and 0.363 in sIgA) was observed. These log-transformed values were significantly and positively correlated (*r* = 0.454, *p* < 0.001). The level of sIL-6 was mildly but significantly correlated with the test time of the saliva sample (rho = 0.174, *p* = 0.0498) (Fig. 1), indicating a trend that later time points had higher sIL-6 levels. The level of sIgA was not correlated with the test time of the saliva sample (*p* = 0.329). Internal consistency for the set of numeric and ordinal items, with item directionality corrected, was acceptable (Cronbach’s α = 0.76).

### Univariate analyses

The results from univariate analyses were summarized in Table 2, identifying several significant factors associated with higher sIL6 levels: living in a multi-generational house (F = 2.994, *p* = 0.021), with post-hoc Tukey analysis indicating significantly higher levels compared to those living with a spouse (*p* = 0.012) and with a partner (*p* = 0.039); higher sIgA (*r* = 0.454, *p* < 0.001); later collection time (rho = 0.174, *p* = 0.0498); non-smoking (t = 2.557, *p* = 0.017); more frequent energy drink consumption (rho = 0.184, *p* = 0.037); higher qualitative job overload (*r* = −0.231, *p* = 0.009); higher physical demands (rho = −0.177, *p* = 0.047); lower vigor (rho = 0.214, *p* = 0.015); greater fatigue (*r* = −0.207, *p* = 0.019); better skill discretion (*r* = 0.177, *p* = 0.047); and higher interoceptive sensibility in not-worrying (*r* = 0.190, *p* = 0.032); and emotional awareness (*r* = 0.221, *p* = 0.012). Several other factors showed trend-level associations.

**Table 2:** Asssociation with log-transformed values of sIL-6 and each factor

Items	statistics	*p*-value	Interpretation	Items	statistics	*p*-value	Interpretation
			Higher sIL6 was associated with…				Higher sIL6 was associated with…
**Stress biomarkers and the test time**				**[Buffering factors]**			
sIgA (log-transformed)	r = 0.454	**<0.001**	higher sIgA	Supervisor support	r = −0.122	0.171	
The test time of the saliva sample	rho = 0.174	**0.0498**	later collection time	Coworker support	r = −0.043	0.630	
**General information**				Support from family and friends	rho = −0.020	0.825	
Age	r = −0.125	0.161		Satisfaction with work and life	r = −0.015	0.870	
Sex	t = −1.607	0.110		**Job Contents Questionnaire**			
Education	rho = 0.094	0.293		Skill discretion	r = 0.177	**0.047**	better skill discretion
BMI	rho = −0.134	0.132		Decision authority	r = 0.060	0.503	
Smoking	t = 2.557	**0.017**	non-smoking	Decision latitude	r = 0.143	0.111	
Marriage status	t = 1.233	0.220		Psychological demand	r = 0.160	0.072	(greater demand)
Home environment	F = 2.994	**0.021**	living in a multi-generational house	Supervisor support	r = −0.056	0.529	
**Life style-related assessments (unstructured)**				Coworker support	r = −0.033	0.710	
**[Dietary and drinking habits]**				Social support	r = −0.051	0.567	
Frequency of diet	t = −0.328	0.744		Demand/Control ratio	r = −0.018	0.837	
Frequency of eating snacks	rho = −0.078	0.379		**Dutch Work Addiction Scale**			
Frequency of eating vegetables	rho = −0.135	0.129		Compulsively	r = 0.109	0.221	
Frequency of drinking (alcohol)	rho = 0.044	0.625		Excessively	r = 0.081	0.366	
Frequency of coffee consumption	rho = 0.024	0.788		Total score	r = 0.105	0.243	
Frequency of energy drink consumption	rho = 0.184	**0.037**	more frequent consumption	Overwork	rho = −0.028	0.764	
Frequency of green tea consumption	rho = −0.164	0.065	(less frequent consumption)	**WHO Health and Work Performance Questionnaire**			
Frequency of red tea consumption	rho = 0.115	0.196		Absolute presenteeism	r = −0.053	0.549	
**[Daily activities]**				Relative presenteeism	rho = −0.096	0.279	
Frequency of exercise	rho = −0.170	0.055	more frequent exercise	**Pittsburgh Sleep Quality Index**			
Time spent using any devices (for personal use)	rho = 0.108	0.224		Global score	r = 0.054	0.543	
Time spent playing games	rho = −0.085	0.338		Subjective sleep quality	rho = −0.077	0.388	
Bath time	rho = 0.065	0.466		Sleep latency	rho = 0.018	0.840	
**Work-related assessments (unstructured)**				Sleep duration	rho = 0.172	0.052	(shorter duration)
**[Position and experience]**				Sleep efficiency	rho = −0.014	0.875	
Job category	F = 0.355	0.840		Sleep disturbance	t = −0.018	0.986	
Job position	F = 0.850	0.469		Use of sleep medication	rho = 0.073	0.419	
Years of employment	r =−0.137	0.122		Daytime dysfunction	rho = 0.008	0.925	
Years of current employment	rho = −0.124	0.162		**Epworth Sleepiness Scale**			
Previous leave of absence	t = −0.371	0.714		Total score	r = −0.118	0.188	
**[Working style]**				**Quick Inventory of Depressive Symptomatology**			
Days of working/week	rho = −0.018	0.840		Total score	r = −0.007	0.939	
Working hours	rho = −0.02	0.821		**Multidimensional Assessment of Interoceptive Awareness**			
Time spent using computers	rho = −0.036	0.685		Noticing	r = 0.013	0.888	
Rest time	rho = −0.131	0.141		Not-distracting	r = 0.000	0.996	
**[Commuting style]**				Not-worrying	r = 0.190	**0.032**	higher interoceptive sensibility
Commuting time	rho = 0.008	0.926		Attention regulation	r = 0.125	0.159	
Days of commuting/week	rho = 0.038	0.672		Emotional awareness	r = 0.221	**0.012**	higher interoceptive sensibility
Telework	t = −0.531	0.597		Self-regulation	r = 0.144	0.105	
**The Brief Job Stress Questionnaire**				Body listening	r = 0.122	0.172	
**[Stressors]**				Trusting	r = 0.158	0.075	(higher interoceptive sensibility)
Quantitative job overload	r = −0.147	0.097	(higher quantitative overload)	**Mindful Attention Awareness Scale**			
Qualitative job overload	r = −0.231	**0.009**	higher qualitative overload	Total score	r = 0.136	0.126	
Physical demands	rho = −0.177	**0.047**	higher physical demands	**Satisfaction With Life Scale**			
Interpersonal conflict	r = −0.009	0.921		Total score	r = 0.044	0.623	
Job control	rho = 0.030	0.740					
Skill utilization	rho = 0.121	0.173					
Suitable job fit	rho = 0.072	0.420					
Meaningfulness of work	rho = 0.168	0.058	(more meaningful)				
**[symptomatic manifestations]**							
Physiological stress reaction	r = −0.025	0.778					
Psychological stress reaction	r = 0.002	0.979					
Vigor	rho = 0.214	**0.015**	greater vigor				
Anger-irritability	r = −0.053	0.550					
Fatigue	r = −0.207	**0.019**	greater fatigue				
Anxiety	r = −0.001	0.995					
Depression	rho = −0.005	0.953					

Bold values indicate potential significance in factors associated with log-transformed values of sIL-6 (*p* < 0.05). Interpretations were provided for items with *p* < 0.1 for the exploratory purpose.

### Multivariable analyses

1. Symptom-based analysis.
  Our model (AIC = 365, Nagelkerke R² = 0.18), adjusted for age, sex, BMI, smoking status, and the timing of saliva sample collection, revealed that higher levels of sIL-6 were significantly associated with relatively higher vigor (β = 0.200, *p* = 0.039) and greater fatigue (β =−0.247, *p* = 0.032) (Table 3). The maximum VIF in this model was 2.23, suggesting a low risk of multicollinearity. The levels of vigor and fatigue were 1.96 ± 0.70 and 2.36 ± 0.73, respectively, indicating lower vigor and greater fatigue compared to the average scores of the Japanese population in both domains (2.26 for vigor and 2.70 for fatigue) [43].

**Table 3:** A symptom-based model with log-transformed values of sIL-6 as a dependent variable

Items	β	*p*-value	Interpretation
			Higher sIL6 was associated with…
**Adjusted factors**			
The time of saliva sample collection	0.140	0.118	
Age	−0.039	0.690	
Female	−0.026	0.899	
BMI	−0.065	0.495	
Smoking	−0.409	0.132	
**Symptomatic manifestations**			
Physiological stress reaction (BJSQ)	0.002	0.985	
Psychological stress reaction (BJSQ)			
Vigor	0.200	**0.039**	greater vigor
Anger-irritability	0.003	0.978	
Fatigue	−0.247	**0.032**	greater fatigue
Anxiety	0.047	0.677	
Depression	0.027	0.833	

Bold values indicate significant factors associated with sIL-6 (*p* < 0.05), even after controlling for multiple potential confounding factors. †These items were recategorized and then included as categorical data in models since the answers have fewer than five levels. *BJSQ *Brief Job Stress Questionnaire, *PSQI *Pittsburgh Sleep Quality Index
2. Stressor-based analysis.
  Our model(AIC = 364, R² = 0.42, maximum VIF = 1.68), adjusted for age, sex, BMI, smoking status, and the timing of saliva sample collection, revealed that higher levels of sIL-6 were significantly associated with higher qualitative job overload (β = −0.222, *p* = 0.029). This significance remains (β = −0.210, *p* = 0.042) in our extended model that includes buffering factors (AIC = 368, R² = 0.24, maximum VIF = 1.82) (Table 4). The score of the qualitative job overload was 1.87 ± 0.45, indicating higher qualitative job overload compared to the average scores of the Japanese population (2.16 for qualitative job overload) [43].

**Table 4:** A stressor-based model with log-transformed values of sIL-6 as a dependent variable

Items	β	*p*-value	β	*p*-value	Interpretation
					Higher sIL6 was associated with…
**Adjusted factors**					
The time of saliva sample collection	0.222	**0.015**	0.222	**0.020**	later collection time
Age	−0.119	0.215	−0.116	0.234	
Female	0.008	0.968	0.009	0.966	
BMI	−0.067	0.495	−0.063	0.528	
Smoking	−0.389	0.160	−0.417	0.139	
**Occupational stressors (BJSQ)**					
Quantitative job overload	0.012	0.906	0.003	0.973	
Qualitative job overload	−0.222	**0.029**	−0.210	**0.042**	higher qualitative job overload
Physical demands^a^	−0.349	0.124	−0.356	0.120	
Interpersonal conflict	−0.073	0.588	0.004	0.981	
Job control	0.184	0.274	0.231	0.185	
Skill utilization^a^	0.065	0.772	0.078	0.731	
Suitable job fit^a^	−0.182	0.417	−0.174	0.456	
Meaningfulness of work^a^	0.138	0.535	0.211	0.357	
**Buffering factors (BJSQ)**					
Supervisor support	-	-	−0.144	0.172	
Coworker support	-	-	−0.051	0.635	
Support from family and friends	-	-	0.048	0.677	
Satisfaction with work and life	-	-	−0.036	0.753	

Bold values indicate significant factors associated with sIL-6 (*p* < 0.05), even after controlling for multiple potential confounding factors. ^a^These items were recategorized and then included as categorical data in models since the answers have fewer than five levels. *BJSQ* Brief Job Stress Questionnaire.
3. Data-driven analysis.
  In our broader model (AIC = 331, R² = 0.42, maximum VIF = 1.54), elevated sIL-6 levels were significantly associated with fatigue (*p* = 0.049), use of energy drinks (*p* = 0.029), living in a multi-generational house (*p* = 0.005), and non-smoking (*p* = 0.014). Additionally, sIL-6 elevation was inversely and dose-dependently associated with green tea consumption (0–1 cup/day: *p* = 0.038 cups; more/day: *p* = 0.046) (Table 5; Fig. 1). Our narrow model (AIC = 334, R² = 0.38, maximum VIF = 1.58) supported these findings.

**Table 5:** A data-driven model with log-transformed values of sIL-6 as a dependent variable

Items	A narrower model		A broader model		Interpretation
	β	*p*-value	β	*p*-value	Higher sIL6 was associated with…
The time of saliva sample collection	0.140	0.079	0.084	0.314	later collection time
Smoking	−0.483	**0.039**	−0.482	**0.041**	non-smoking
Living in a multi-generational house	0.735	**0.001**	0.644	**0.004**	living in a multi-generational house
Use of energy drink	0.432	**0.010**	0.464	**0.012**	more frequent consumption
Green tea habits (ref. none)					
- 0–1 cup/day			−0.423	**0.048**	less frequent consumption
- 2 cups and more/day			−0.590	**0.028**	
Exercise (once a week or more)			−0.082	0.634	
Quantitative job overload (BJSQ)			0.103	0.257	
Qualitative job overload (BJSQ)	−0.078	0.346	−0.136	0.137	
Physical demand (BJSQ)^a^	−0.207	0.287	−0.117	0.559	
Meaningfulness of work (BJSQ)^a^			0.055	0.753	
Vigor (BJSQ)	0.042	0.639	0.078	0.408	
Fatigue (BJSQ)	−0.239	**0.005**	−0.230	**0.010**	greater fatigue
Skill discretion (JCQ)	0.069	0.419	0.064	0.456	
Short sleep duration (PSQI)^a^			0.313	0.115	
PCA-derived interoceptive component (MAIA)	0.250	**0.007**	0.166	**0.024**	higher interoceptive sensibility

Bold values indicate significant factors associated with sIL-6 (*p* < 0.05). ^a^These items were recategorized and then included as categorical data in models since the answers have fewer than five levels. *BJSQ *Brief Job Stress Questionnaire, *JCQ *Job Contents Questionnaire, *MAIA *Multidimensional Assessment of Interoceptive Awareness, *PSQI *Pittsburgh Sleep Quality Index

## Discussion

To the best of our knowledge, this is the first study to reveal factors associated with inflammation as a stress response among stressed office workers using objective stress markers, showing an association between elevated sIL-6 levels and symptomatic manifestations, particularly increased fatigue and relatively higher vigor. Notably, higher qualitative job overload emerged as the most influential stressor related to sIL-6 levels. We also explored potential lifestyle-related associations and found that elevated sIL-6 was associated with multi-generational households and greater consumption of energy drinks but was inversely associated with that of green tea. These factors may act as confounders beyond those directly related to occupational stress. Our data-driven model, which included these confounders, supported fatigue as a symptomatic manifestation reflecting inflammation. It should be noted, however, that causal relationships cannot be inferred due to the cross-sectional nature of the study. A trend that later time had higher sIL-6, which was statistically adjusted, partially aligns with an existing report showing elevated levels in the evening [28], but the timing of the minimum point differed, as the prior report identified 1 pm. as the lowest point, whereas our results showed lower levels at earlier time points. The positive relationship between sIL-6 and sIgA levels suggests that the inflammatory response remains preserved in this sample, despite presumably prolonged occupational stress exposure. This might reflect their condition in which participants though at high risk, have not yet developed clinical conditions. It could also highlight the potential utility of sIL-6 in the direct and early detection of stressed and “inflamed” states that heighten the risk of both physical and mental disorders.

Our finding of a significant relationship between sIL-6 levels and fatigue among stressed office workers highlights the importance of monitoring fatigue levels to detect inflammation and facilitate early intervention in this population. This result aligns closely with findings from various studies in other populations, including those with cancer-related fatigue [38], long-COVID [43], COVID-19 vaccinated individuals [42], chronic hemodialysis [41], diabetes [35], stroke survivors [37], Parkinson’s disease [40], multiple sclerosis [36], and rheumatoid arthritis [39]. Although fatigue is an indistinguishable depressive symptom in patients with depression, high serum IL-6 levels have been observed in various types of depression [63, 64]. A prospective, large-scale cohort study demonstrated a significant association between new-onset fatigue and high IL-6 [33]. Several interventional studies have suggested a causal relationship between IL-6 and fatigue [24, 65, 66]. Administration of IL-6 in healthy individuals promotes fatigue and alters sleep structure [65], while IL-6 inhibitors in patients with rheumatoid arthritis reduce the level of fatigue, which precedes the anti-inflammatory effects on joint disease, suggesting a direct effect of IL-6 on the central nervous system [66].

Relatively higher vigor associated with elevated sIL-6 levels may reflect stress-related activation to address their occupational stress. Importantly, our participants exhibited reduced vigor levels compared to the general population. Within this overall reduction, the observed relatively preserved vigor may result from activation of the sympathetic nervous system and the HPA axis as excessive stress responses, potentially masking an underlying state of exhaustion. While such activation may be adaptive in the context of acute stress, its persistence under chronic conditions becomes maladaptive. However, such apparent vigor during prolonged stress may carry an increased risk of burnout, adjustment disorder, and depression [13, 67].

We found that qualitative job overload, rather than quantitative, was the most influential stressor associated with sIL-6 levels. Qualitative job overload refers to the cognitive demands of work that require intense concentration and advanced knowledge or skills, whereas quantitative job overload reflects a large volume of tasks. This distinction may indicate that cognitive burden plays a more central role in stress responses among workers. Notably, certain cognitive tasks used in experimental stress induction, such as mental arithmetic, have been shown to trigger inflammatory responses, although this may reflect a non-specific reaction across various stress types [68, 69]. Our findings may be influenced by the characteristics of our sample, i.e. office workers, and may also reflect the growing qualitative demands driven by the rapidly evolving industrial structure of modern society [70]. This suggests that addressing qualitative job overload in contemporary office work settings may be a key strategy for preventing inflammation-related health risks, although this does not imply that other forms of stress should be overlooked.

Beverage consumption habits may be important for managing bodily stress responses because they are noticeable and modifiable. Energy drink use is associated with higher sIL-6. One interpretation is that energy drinks can exaggerate the bodily stress response via the activation of the HPA axis and sympathetic nervous system [71]. Boosting stress responses can be maladaptive, particularly during long-term stress exposure, finally resulting in persistent bodily stress responses, including elevated sIL-6 levels. Another interpretation is that the frequent use of energy drinks may be reflected in the need for stimulants to address ongoing stress and daytime sleepiness, with elevated sIL-6 levels as a potential consequence of chronic stress and insufficient sleep. Elevated sIL-6 levels and poor sleep quality could mutually exacerbate each other [23, 72]. Short- and long-term strategies are required to address occupational stress and treat low-grade systemic inflammation.

In contrast, a more frequent habit of drinking green tea was associated with lower sIL-6 levels. The effects of green tea consumption on sIL-6 levels may be categorized into direct effects (anti-inflammatory properties) and indirect effects, such as relaxing effects, which lead to deactivation of the HPA axis and sympathetic nervous system [73–75]. Although the preventive effect of green tea consumption on depression, commonly observed in occupationally stressful environments, remains inconsistent, possibly because of its indirect and complex relationship [76, 77], our findings suggest that green tea could help reduce stress reactions in such settings. However, as this is a cross-sectional study, causality cannot be determined.

The stress stemming from living in a multi-generational household may be outside of occupational stress but should not be underestimated. While various factors related to a multi-generational household, such as social support and financial burden, could contribute to its significance, given the age of our sample and the lack of significance in other categories, such as having children, caregiving for parents might be a plausible and noteworthy stressor, along with complex domestic interpersonal relationships. Caregiving is a well-known stressor associated with various mental illnesses, such as depression, and its role in inducing inflammation has been demonstrated [78, 79]. The necessity for continuous stress responses, even after work, can exacerbate the consequences of long-term occupational stress [80].

The item of MAIA should be carefully interpreted because a higher score typically indicates a beneficial aspect, reflecting a balanced approach that avoids unnecessary concern over the sensations of pain or discomfort [56]. This result does not align with our initial expectation, and given the limitations of self-reported measures, further investigation is warranted. One possible explanation for this unexpected finding is that strong internal bodily signals (e.g., cardiac, hormonal, inflammatory) within our sample may influence this outcome [61, 81]. Furthermore, in a stressed population, not-worrying could represent a misinterpretation of these bodily alert signals, potentially resulting in inadequate stress management. This may highlight the importance of accurate self-monitoring of bodily conditions. One study showed that low-grade inflammation, as assessed by CRP, was associated with a high score of not-worrying in MAIA [82], although further research is required to investigate this association and the reasons behind it.

The finding of lower sIL-6 levels in smokers should be interpreted with caution, as smoking can induce local inflammation and activate both the sympathetic nervous system and the HPA axis, typically leading to elevated sIL-6 levels [83], despite reports of its complex effects on inflammation [84]. One possible explanation may relate to our specific sample, which may already exhibit prolonged activation of these systems, potentially leading to a blunted inflammatory response due to chronic physiological adaptation. Moreover, smoking timing was not controlled in our study, potentially influencing the results. Importantly, this finding should not be interpreted as evidence of benefit from smoking, but rather highlights the need for further studies to elucidate its impact on stress-related inflammatory responses.

The discrepancy between our inclusion criteria and our findings may instead highlight the importance of monitoring symptomatic manifestations as stress responses, rather than focusing solely on external stressors, in managing stress within this population. Although we observed a trend suggesting that sIL-6 was associated with quantitative job overload, which partially supports our inclusion criteria for selecting stressed workers, this discrepancy may be attributed to the inherent limitations of self-reported measures, as well as a conceptual distinction between stressors (e.g., job demands, control) and stress responses (e.g., inflammation). Given that chronic exposure to stressors induces prolonged stress responses, which may ultimately lead to mental health problems, stress responses could serve as more direct and sensitive indicators of mental health risk than stressors themselves. However, further longitudinal research is required to confirm this.

One strength of this study is that it examines associations between self-perceived stress-related constructs and a biological marker. Another strength is the inclusion of a large number of specific samples, that is, stressed office workers, which enhanced the sensitivity to detect direct relationships, thereby avoiding general gradational relationships that stem solely from overall severity. However, this study has certain limitations. First, the causal direction cannot be inferred from statistical associations, given the cross-sectional design. Second, we did not assess other stress responses, such as cortisol levels. Because strict control of saliva sampling time was not feasible in our study setting, we selected IL-6 and sIgA as biomarkers, given their relatively moderate diurnal variability compared to cortisol within our sampling timeframe [28, 85]. However, this choice limits a comprehensive understanding of bodily stress responses. Third, sIL-6 levels can be influenced by various forms of acute stress, as well as chronic occupational stress. Although this research was conducted under strict infection control due to the COVID-19 era, the possibility of asymptomatic infections could not be completely ruled out. However, no infections were reported among the research staff following participant contact. Fourth, although saliva samples were collected between 10 a.m. and 5 p.m., and the sampling time was statistically controlled, the exact timing of collection and participants’ conditions, such as their fasting status and recent physical activity, were not strictly standardized. Fifth, the characteristics of our sample, such as Japanese office workers, may limit the generalizability. In addition, participants who were able to take part in this study despite being under occupational stress may be unique, further restricting the generalizability of our findings. Sixth, while focusing on stressed workers enhanced sensitivity in detecting the direct relationship between sIL-6 levels and their characteristics, avoiding a general gradational relationship driven solely by overall severity, we could not provide any evidence for less stressed workers. Further studies are required to address these limitations. Related to this, our recruitment method based on general population norms may carry a risk of bias. In particular, individuals who exhibit low questionnaire scores but high levels of inflammation, potentially indicating poor insight, may have been excluded. This represents a possible source of selection bias. Seventh, all questionnaires used are self-report instruments and therefore subject to inherent biases. Eighth, stress-related questionnaires that more directly assess perceived stress reactivity, such as the Perceived Stress Scale [86] and the Ford Insomnia Response to Stress Test [87], were not included in this study due to time constraints. Future research is needed to examine this aspect for a more comprehensive understanding. Ninth, despite a relatively large number of samples, the sample size may still be insufficient to confirm our findings given the number of variables included in the analysis. Further studies with larger sample sizes are warranted.

## Conclusions

While this study has important limitations, such as the reliance on self-report instruments and the limited generalizability to broader working populations, our findings suggest that the feeling of fatigue may reflect chronic stress-induced systemic low-grade inflammation in the body, highlighting the importance of self-monitoring fatigue for early intervention. Inflammation-based stress management holds promise in preventing both mental and physical illnesses in stressed office workers.

## Data Availability

The datasets generated during the current study are available from the corresponding author upon reasonable request and subject to regulatory approval.

## Abbreviations

BJSQ: Brief Job Stress Questionnaire
CRP: C-reactive protein
DUWAS: Dutch Work Addiction Scale
ESS: Epworth Sleepiness Scale
HPA: Hypothalamic–pituitary–adrenal
JCQ: Job Content Questionnaire
MAAS: Mindful Attention Awareness Scale
MAIA: Multidimensional Assessment of Interoceptive Awareness
PSQI: Pittsburgh Sleep Quality Index
QIDS: Quick Inventory of Depressive Symptomatology
sIgA: Salivary immunoglobulin A
sIL-6: Salivary interleukin-6
SWLS: Satisfaction with Life Scale
WHO-HPQ-SF: World Health Organization – Health and Work Performance Questionnaire
